# Neurofibromin knockdown in glioma cell lines is associated with changes in cytokine and chemokine secretion *in vitro*

**DOI:** 10.1038/s41598-018-24046-2

**Published:** 2018-04-11

**Authors:** Matthew D. Wood, Joydeep Mukherjee, Russell O. Pieper

**Affiliations:** 1 Department of Pathology, Division of Neuropathology, University of California San Francisco, San Francisco, CA USA; 2 Department of Neurological Surgery, University of California San Francisco, San Francisco, CA USA; 3 Present Address: Department of Pathology, Oregon Health & Science University, Portland, OR USA

## Abstract

The neurofibromin-1 tumor suppressor gene (*NF1*) is altered in approximately 20% of sporadic glioblastoma (GBM) cases. *NF1* deficient GBM frequently shows a mesenchymal gene expression signature, suggesting a relationship between *NF1* status and the tumor microenvironment. To identify changes in the production of secreted cytokines/chemokines in *NF1* deficient glioma, we applied cytokine arrays to conditioned media from a panel of three GBM cell lines after siRNA-mediated *NF1* knockdown. We identified increased secretion of platelet-derived growth factor AA (PDGF-AA), chitinase-3-like protein 1 (CHI3L1), interleukin-8 (IL-8), and endoglin (ENG) in different subsets of these cell lines. Secretion was associated with induction of the corresponding messenger RNA, suggesting a mechanism involving transcriptional upregulation. By contrast, in non-transformed immortalized normal human astrocytes, PDGF-AA secretion was increased upon *NF1* knockdown, while secreted CHI3L1, ENG, and IL-8 were reduced or unchanged. Analysis of The Cancer Genome Atlas confirmed a relationship between glioma *NF1* status and *ENG* and *CHI3L1* in tumor samples. Overall, this study identifies candidate changes in secreted proteins from *NF1* deficient glioma cells that could influence the tumor microenvironment, and suggests a direct link between *NF1* loss and increased tumor cell production of CHI3L1 and endoglin, two factors implicated in mesenchymal identity in glioblastoma.

## Introduction

Mutation in the *NF1* gene cause neurofibromatosis type 1 (NF1), which predisposes patients to the development of peripheral and central nervous system (CNS) tumors, along with several other clinical manifestations. The most common CNS neoplasm in the setting of NF1 is a WHO grade I pilocytic astrocytoma. However, NF1 patients are also predisposed to developing higher-grade infiltrating gliomas^1^. In 2008, The Cancer Genome Atlas (TCGA) analysis of glioblastoma (GBM) revealed *NF1* mutations or other alterations in approximately 20% of sporadic GBM, i.e. in patients without NF1^2^. Since this discovery, there has been considerable interest in identifying a role for *NF1* alterations in sporadic GBM.

*NF1* encodes neurofibromin, a GTPase activating protein that serves as a negative regulator of Ras signaling and Ras-associated downstream signaling pathways^3^. In NF1, several tumor types have been shown to have inactivation of the wild-type *NF1* allele, supporting the notion that *NF1* is a bona fide tumor suppressor gene^4,5^. In sporadic glioblastoma, a post-translational mechanism for neurofibromin degradation has also been described^6^. Compared to diffusely infiltrating gliomas with intact *NF1*, lower grade gliomas with *NF1* alteration have a worse prognosis, while GBM with *NF1* alteration has a similar clinical outcome to cases without *NF1* alterations^7^.

Gene expression profiling of GBM has shown that *NF1* alterations are associated with a so-called mesenchymal gene expression pattern^8,9^. Recent data suggest that the mesenchymal gene expression pattern may reflect features in the tumor microenvironment, rather than an intrinsic feature of the neoplastic cells^10,11^. This association between *NF1* loss and the mesenchymal gene expression pattern suggests a direct role for GBM *NF1* status influencing the tumor microenvironment. Supporting this possibility, numerous inflammatory mediators–including some factors that are regulated by signaling pathways downstream of Ras, such as MEK-ERK and Akt–have been implicated in GBM initiation and progression^12^. Tumor *NF1* status strongly predicts the presence of tumor-infiltrating lymphocytes, and a recent mechanistic study showed that conditioned media from *NF1*-deficient glioma cells increases macrophage recruitment *in vitro*^10,13^.

In order to study the role of *NF1* as a mediator of the tumor microenvironment in GBM, we examined how *NF1* loss influences secreted factors from neoplastic cells by applying glioma cell line conditioned media to cytokine arrays. Surprisingly, only a few recurrent changes were identified across a panel of three glioma cell lines. Here, we report that neurofibromin knockdown was associated with increases in expression and secretion of platelet-derived growth factor AA (PDGF-AA) and interleukin-8 (IL-8), two factors that influence tumor angiogenesis and inflammatory cell recruitment, and chitinase-3-like protein 1 (CHI3L1) and endoglin (ENG), two markers of the mesenchymal gene expression subclass in GBM. Correlation of our *in vitro* finding with TCGA data suggests an *in vivo* association between *NF1* status and tumor production of CHI3L1 and ENG, supporting an association between *NF1* and mesenchymal identity in human GBM samples.

## Methods

### Cell lines

Normal human astrocytes immortalized by expression of the catalytic subunit of telomerase and the E6/E7 oncoproteins were generated in our laboratory and have been previously described^14^. T98G cells were obtained from American Type Culture Collection (ATCC). SF268 and SF295 cells were provided by the University of California San Francisco (UCSF) Brain Tumor Research Center Tissue Core. Media was obtained from the UCSF Cell Culture Facility. Cell lines were maintained at 37 °C in a humidified 5% CO_2_ incubator in high glucose Dulbecco’s modified Eagles media (DMEM-H21) supplemented with 10% fetal bovine serum (FBS), 1% penicillin/streptomycin, and 1% fungizone. All cell lines were negative for mycoplasma by PCR analysis (forward primer GPO3 5′-GGGAGCAAACAGGATTAGATACCCT-3′, reverse primer MGSO 5′-TGCACCATCTGTCACTCTGTTAACCTC-3′)^15^. Cell line identities were confirmed by short tandem repeat analysis.

### siRNA Transfection

ON-TARGETplus siRNA SMARTpools were purchased from Dharmacon (Lafayette, CO) and reconstituted according to the manufacturer’s instructions. Cells were seeded at 0.1 × 10^6^ cells per well in triplicate wells of 6-well tissue culture plates in DMEM-H21 10% FBS without antibiotics. Twenty-four hours later, cells were transfected with 20 nM siControl (product number D-001206-14-05) or siNF1 (product number L-003916-00-005) SMARTpools, using DharmaFECT 1 reagent according to the manufacturer’s protocol. Eighteen hours after transfection, the transfection medium was replaced with DMEM 10% FBS with antibiotics, and cells were incubated for a total of 72 hours from initial transfection before harvesting the conditioned media for ELISA, and the cells for total RNA and protein extraction.

### Western Blotting

Cells were harvested by washing once with ice-cold phosphate buffered saline (PBS), then scraping into ice-cold PBS, followed by centrifugation for 3 minutes at 200 × g and resuspension in lysis buffer (20 mM Tris-HCL pH 7.4, 100 mM NaCl, 300 mM sucrose, 6 mM MgCl_2_, 1 mM EGTA, 0.5% Triton X-100) supplemented with 1 × PhosStop/protease inhibitor cocktail (Sigma-Aldrich, St. Louis, MO). Protein was extracted by overnight incubation at 4 °C with gentle rotation, followed by clarifying lysates by centrifugation at 16,000 × g at 4 °C for 10 minutes. Protein concentrations were determined by DC Protein Assay (Bio-Rad Laboratories, Hercules, CA). Total protein (40 ng) in 1 × loading dye with 0.1 M DTT was fractionated on Novex 4%–20% Tris-Glycine gels (Invitrogen, Carlsbad, CA) for 2 hours at 100 volts, then transferred to Immuno-Blot PVDF membranes (Bio-Rad Laboratories) overnight at 4 °C for 18 hours in transfer buffer without methanol. Membranes were blocked for 1 hour in 5% nonfat milk (Bio-Rad Laboratories) suspended in 1 × tris-buffered saline with 0.05% Tween-20 (TBST; Teknova, Hollister, CA). Primary antibodies were diluted in 1 × TBST 5% milk or 5% bovine serum albumin (BSA, Sigma-Aldrich, St. Louis, MO) and applied for 18 hours at 4 °C with gentle rocking. Membranes were washed 3 times quickly and then 3 times for 15 minutes in 1 × TBST at room temperature before applying HRP-conjugated secondary antibodies diluted 1:20,000 in 5% milk TBST (anti-mouse HRP sc-2031 and anti-rabbit HRP sc-2030 from Santa Cruz Biotechnologies, Dallas, TX). After repeating the 1 × TBST wash steps, bands were detected by ECL reagent (GE Healthcare, Chicago, IL). Primary antibodies were obtained from Cell Signaling Technology (Danvers, MA), ERK #9102, diluted 1:2000 in 5% BSA 1 × TBST, phospho-ERK #9106, diluted 1:2000 in 5% milk 1 × TBST, and GAPDH #2118, diluted 1:5000 in 5% BSA 1 × TBST, or Santa Cruz Biotechnologies, NF1 H-12 mouse monoclonal sc-376886 diluted 1:100 in 5% milk 1 × TBST.

### Cytokine Array

Cells were transfected as described in the siRNA protocol, except the medium was replaced with serum- and antibiotic-free DMEM-H21 for the final 24 hours of the incubation. Medium from 3 wells was pooled, clarified by centrifugation at 200 × g at 4 °C, and immediately applied to Proteome Profiler Human XL cytokine arrays according to the manufacturer’s instructions (R&D Systems, Minneapolis, MN). Cytokine array signal was detected at multiple exposure times ranging from 15 seconds to 10 minutes. Film was scanned using an Epson Perfection V78 Pro transmission mode scanner and Epson SilverFast software. Signal levels were measured using ImageJ software with the Protein Array Analyzer plugin^16^. Values from duplicate spots were averaged, and the relative signal was calculated in siNF1 treated sample, compared to siControl treated cells.

### Quantitative RT-PCR

When cells were harvested for protein extraction, an aliquot of the PBS resuspended cells was removed, centrifuged at 300 × g for 5 minutes, and cell pellets were stored at −80 °C. Total RNA was extracted with RNEasy Mini Kit (Qiagen, Hilden, Germany) and cDNA was synthesized from 1000 ng of total RNA in a 40 μL reaction using Supertscript II Reverse Transriptase (Invitrogen) and oligo-dT primers from Integrated DNA Technologies (Coralville, IA). 1 μL of the cDNA reaction was used for quantitative RT-PCR using the Rotor-Gene System (Qiagen) and the following qPCR primers obtained from Integrated DNA Technologies: *HPRT1* (forward 5′-GGTCAGGCAGTATAATCCAAAGA-3′, reverse 5′-GAAGCGGTCAAGGGCATCT-3′), *NF1* (forward 5′-GGACTCTAAGATCAACACCCTG-3′, reverse 5′-CACCACACTCTGCACAATTCCAT-3′, reference^17^), *CHI3L1* (forward 5′-GTGAAGGCGTCTCAAACAGG-3′, reverse 5′-GAAGCGGTCAAGGGCATCT-3′), *PDGFA* (forward 5′-GCAAGACCAGGACGGTCATTT-3′, reverse 5′-GGCACTTGACACTGCTCGT-3′), *CXCL8/IL8* (forward 5′-GCTCTGTGTGAAGGTGCAGT-3′, reverse 5′-ACTTCTCCACAACCCTCTGC-3′), and *ENG* (forward 5′-TGCACTTGGCCTACAATTCCA-3′, reverse 5′-AGCTGCCCACTCAAGGATCT-3′). Some qPCR primers were picked using the PrimerBank qPCR primer database^18^. Reaction conditions were as follows: 95 °C for 10 minutes, 40 cycles of 95 °C 10 seconds, 54 °C 15 seconds, and 72 °C 20 seconds). Differences in RNA expression between siNF1 and siControl treated cells was determined by the ΔΔCt method^19^.

### ELISA

Medium from three wells of siRNA treated cells was pooled and clarified by centrifugation at 200 × g for 5 minutes at 4 °C, and aliquots were stored at −20 °C. ELISA for secreted factors was performed using kits, according to the manufacturer’s instructions (CHI3L1 product MC3L10 from R&D Systems, PDGF-AA product ab100622 from Abcam [Cambridge, United Kingdom], endoglin product DNDG00 from R&D Systems, and IL-8 product D8000C from R&D Systems). Conditioned medium from three independent transfections was analyzed.

### TCGA Analysis

Data were retrieved from the cBioPortal database in August 2017. Glioblastoma TCGA data for 291 complete samples was queried for mutations, copy number alterations (GISTIC), and RNA expression z-scores (RNASeq V2 RSEM, threshold +/− 2) using *NF1*, *CHI3L1*, *PDGFA*, *ENG*, and *CXCL8* as the input gene set^2^. Overall, 147 cases had RNA expression data. *NF1*-altered cases (mutation, mRNA downregulation > 2-fold, or homozygous deletion, N = 12) were compared to cases with no *NF1* alteration (N = 129). Cases with *NF1* mRNA upregulation (i.e. mRNA z-score > 2, N = 6) were excluded from the analysis.

### Statistical Analysis

Data analysis was performed using GraphPad Prism software. Graphs of ELISA and qPCR data show the mean ± standard deviation of three independent experiments. Significance levels are from paired bidirectional T-tests, with *p < 0.05, **p < 0.01, and ***p < 0.001. Statistical tests on the qPCR data for each cell line were adjusted for multiple comparisons (i.e. 4 comparisons for each cell line). Gene expression z-scores from TCGA were analyzed by Mann-Whitney test, also using GraphPad software.

### Data Availability Statement

All data generated or analyzed during this study are included in this published article, and its Supplementary Information files.

## Results

**Figure 1:**
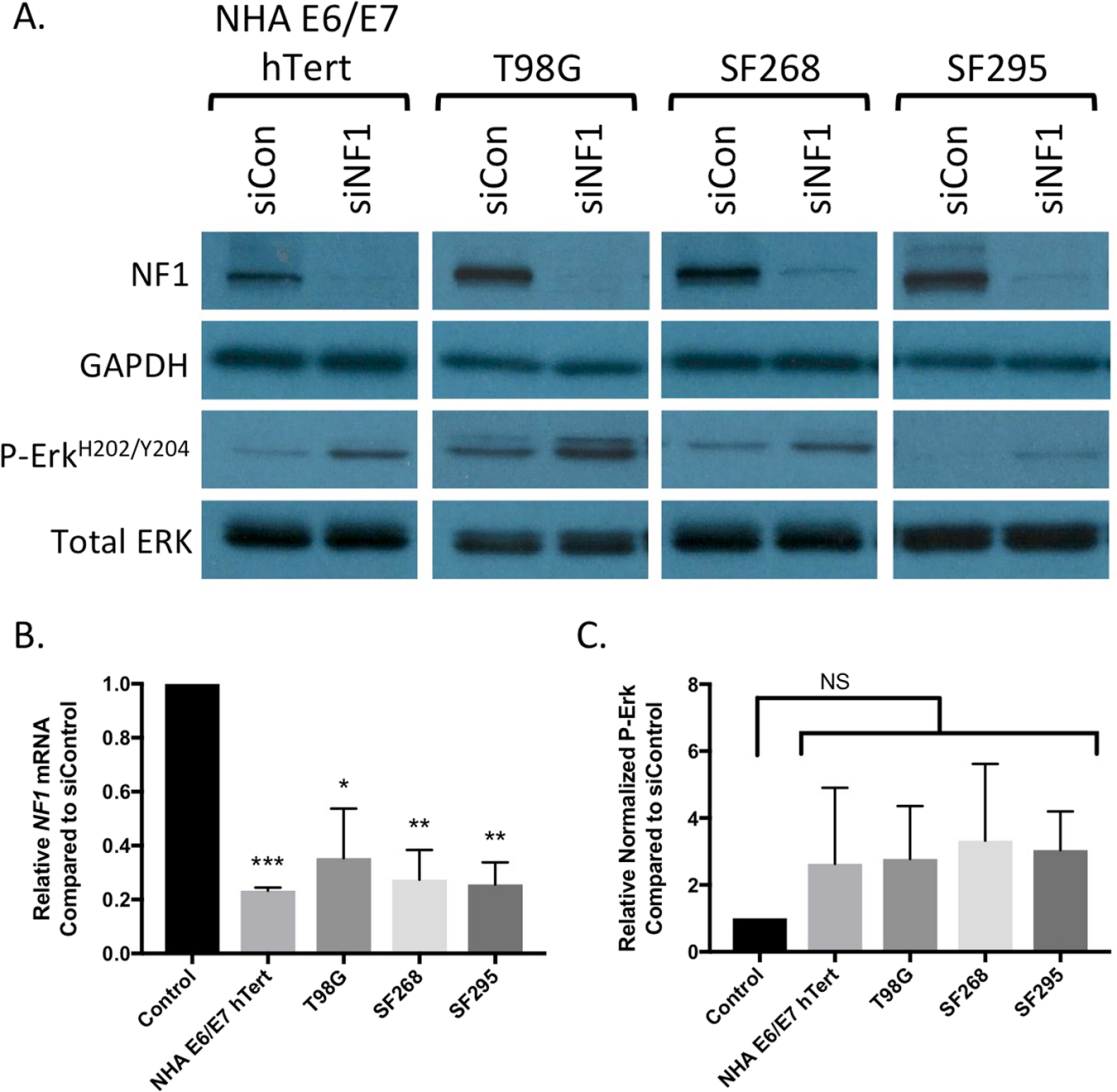
Confirmation of neurofibromin knockdown by Western blot (**A**) and quantitative RT-PCR (**B**) in four cell lines used in the study. All cell lines showed an increase in phosphorylated ERK protein by Western blot (**A**). (**C**) Quantitation of the increased phospho-ERK signal did not achieve statistical significance (values are mean +/− standard deviation of 3 independent replicates for NHA, T98G, and SF268, and of 2 replicates for SF295; in a third SF295 assay, P-Erk levels were too low for reliable quantitation).

To establish conditions for knocking down neurofibromin protein, we treated three glioblastoma cell lines (T98G, SF268, and SF295) and non-transformed immortalized normal human astrocytes (NHA-E6/E7-hTert) with a SMARTpool of four siRNA constructs targeting the *NF1* transcript (siNF1) optimized for target specificity, or a nontargeting SMARTpool control (siControl). According to the canSAR database, T98G, SF295, and SF268 do not have any *NF1* mutations, and all of the cell lines express neurofibromin protein, therefore the cell lines that we selected for this study are highly likely to be neurofibromin proficient at baseline^20,21^. Seventy-two hours after the siRNA transfection, we observed a statistically significant reduction in *NF1* messenger RNA across all four cell lines, and an associated reduction in detectable neurofibromin protein by Western blot (Fig. 1A,B; cropped images are shown to conserve space, and the expanded Western blots and additional information on the Western blotting methods are provided in Supplemental Figure S1). This was associated with a detectable increase in ERK phosphorylation across all cell lines, as assessed by Western blot with phospho-specific antibodies (Fig. 1A). Upon quantitation, this trend this did not reach statistical significance due to high variability between replicates (Fig. 1C). None of the cell lines showed induction of p21 (data not shown), a senescence marker that can be induced by strong *NF1* knockdown as a result of oncogene-induced senescence in some glioma cell lines^22^.

**Figure 2:**
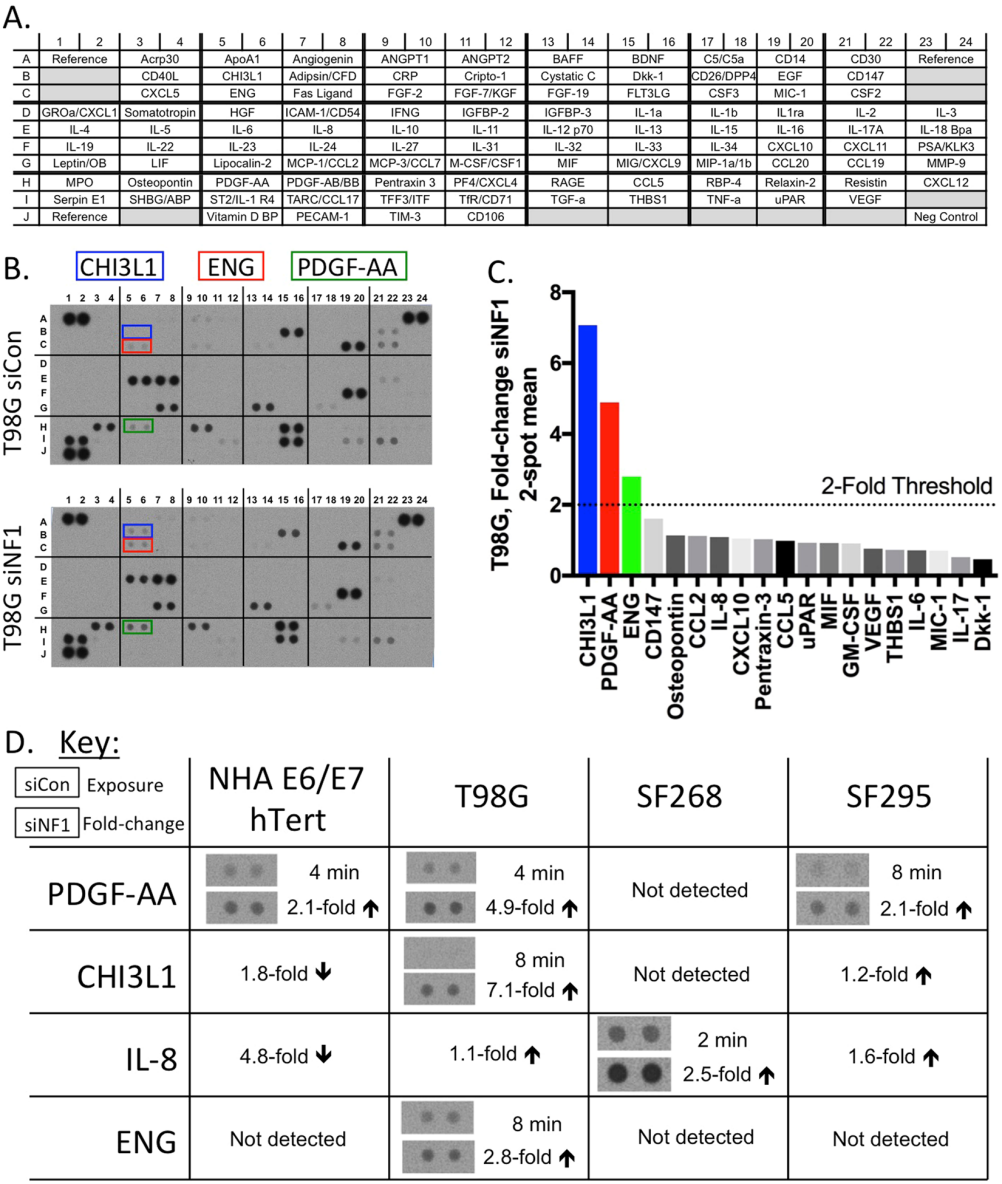
Human cytokine array results from conditioned media of four cell lines after neurofibromin knockdown. (**A**) Reference key for cytokine array, adapted from the manufacturer’s information. (**B**) Complete array images and (**C**) quantitation of resulting data from siControl and siNF1 treated T98G cells. Quantitation was performed on a 2 minute exposure, except for CHI3L1 (8 minute exposure). (**D**) Summary of array data for all factors with changes upon siNF1 treatment. Only factors with at least 2-fold increased expression upon NF1 knockdown are pictured, other changes are noted by text only. Complete array images and quantitations are provided in the supplementary information.

We collected conditioned media from siControl or siNF1 transfected cells and applied the media to protein arrays that detect 105 human cytokines and chemokines (Fig. 2A). An example of the data generated by this analysis is shown in Fig. 2B,C for T98G cells, and the complete findings are summarized in Fig. 2D and Supplemental Figure S2. Quantitation of the relative signal intensity of siNF1 versus siControl conditioned medium showed a greater than 2-fold increase in secreted PDGF-AA in T98G, SF295, and NHA-E6/E7-hTert cell lines. Secreted CHI3L1 was increased by 7-fold in T98G and by 1.2-fold in SF295. Secreted IL-8 was increased by 2.5-fold in SF268 cells, and by 1.6-fold in SF295 cells. Secreted ENG was increased only in T98G cells, by 2.8-fold. The cytokine array results show that under *in vitro* conditions, knockdown of neurofibromin protein is associated with relatively few changes in secreted cytokines and chemokines, with a few common alterations in secreted proteins across the examined glioma cell lines.

**Figure 3:**
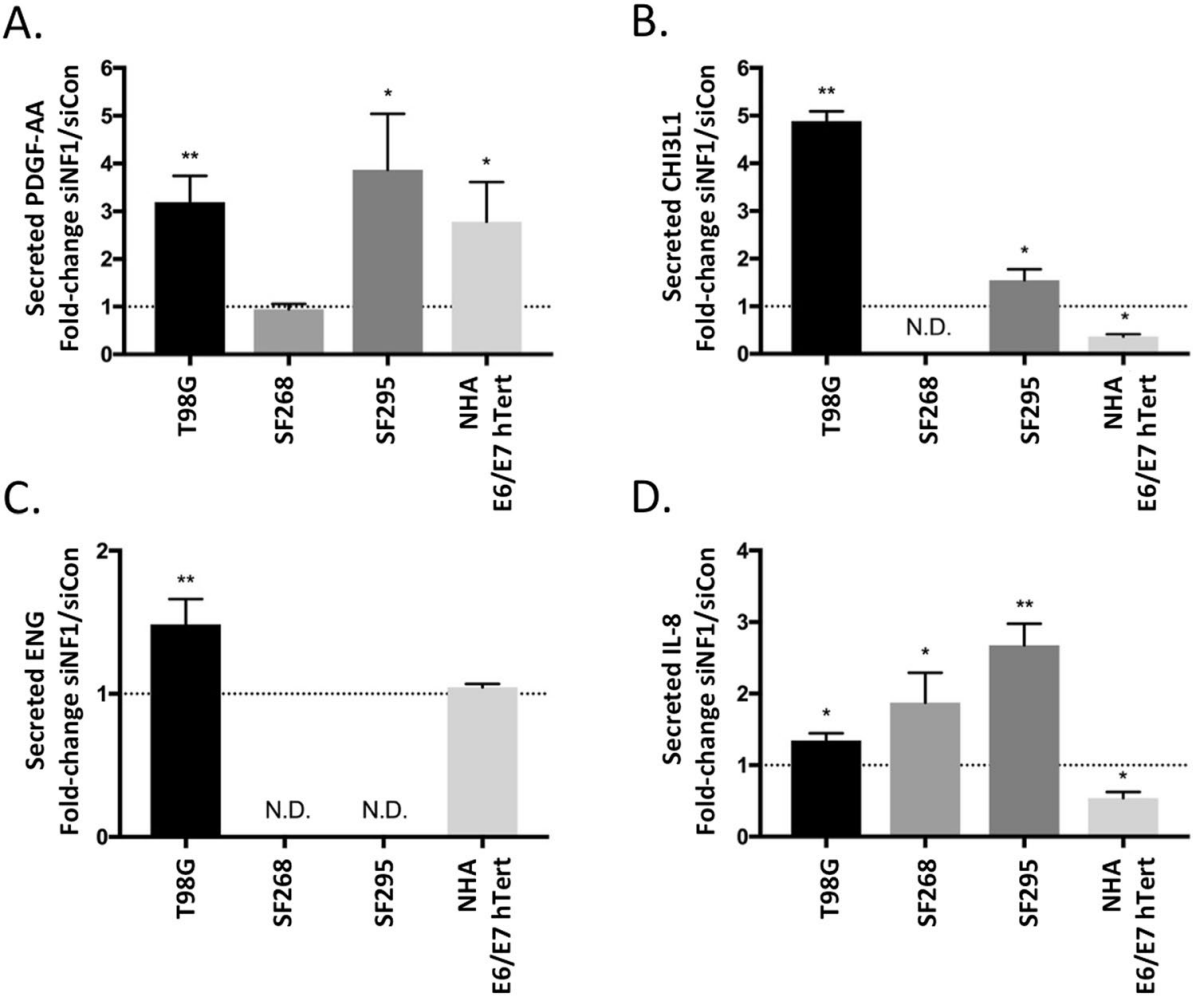
ELISA confirmation of factors increased on cytokine array for (**A**) secreted PDGF-AA, (**B**) secreted CHI3L1, (**C**) secreted ENG, and (**D**) secreted IL-8. Graphs represent the mean fold-change +/− standard deviation of three replicates. N.D. indicates that the factor was not detected.

To confirm the cytokine array findings, we tested clarified conditioned media from an independent set of triplicate samples of conditioned media from siControl or siNF1 treated cells by ELISA. We analyzed all four cell lines for changes in secreted CHIL3L1, PDGF-AA, IL-8, and ENG, since these were the most consistently and/or significantly altered factors identified by the cytokine array analysis. The measured secreted cytokine values are listed in Table 1, and relative changes are shown in Fig. 3 and described here. Consistent with the cytokine array results, we detected an increase in secreted PDGF-AA in *NF1* deficient T98G, SF295, and NHA-E6/E7-hTert cells, while secreted PDGF-AA was unchanged in SF268 (Fig. 3A). Also consistent with the cytokine array results, we observed increased secreted CHI3L1 from siNF1 transfected T98G cells, and an increase in secreted CHI3L1 from siNF1 transfected SF295 cells above a high baseline level of secretion in the control condition (Fig. 3B, and Table 1). Secreted CHI3L1 was reduced by siNF1 treatment in NHA-E6/E7-hTert (Fig. 3B), and was not detectable in SF268 conditioned media under either siRNA condition. Secreted ENG was increased in T98G cells and unchanged in NHA-E6/E7-hTert, but was not detected in SF268 or SF295 cells (Fig. 3C). ELISA for IL-8 was complicated by high inter-replicate variability in the absolute measured IL-8 levels, possibly related to storage of the conditioned media, therefore the measured values in Table 1 are from 2 (rather than 3) experimental replicates. However, when the IL-8 levels were expressed as a ratio of siNF1 to siControl levels across all three replicates, there was a consistent induction of secreted IL-8 in T98G, SF268, and SF295 cells, while in NHA-E6/E7-hTert the secreted IL-8 level was significantly reduced (Fig. 3D), closely paralleling the findings from the cytokine arrays. Taken together, the ELISA data confirm the cytokine array findings and implicate PDGF-AA, CHI3L1, IL-8, and ENG as secreted factors under negative regulatory control by neurofibromin in a subset of glial tumor cell lines *in vitro*.

**Table 1:** Measured concentrations of secreted PDGF-AA, CHI3L1, ENG, and IL-8 in conditioned media from siControl and siNF1 treated cell lines. Values are from three independent replicates, except for IL-8 (2 replicates).

Factor	Cell Line	Mean ± SD (pg/mL)	
		siCon	siNF1
PDGF-AA	T98G	1691 ± 369	5035 ± 468
	SF268	1439 ± 422	1327 ± 255
	SF295	797 ± 185	2995 ± 751
	NHA E6/E7 hTert	3014 ± 1246	7697 ± 707
CHI3L1	T98G	133 ± 69	640 ± 305
	SF268	*Not detected*	*Not detected*
	SF295	13180 ± 1919	20146 ± 1322
	NHA E6/E7 hTert	780 ± 308	273 ± 80
ENG	T98G	455 ± 140	591 ± 125
	SF268	*Not detected*	*Not detected*
	SF295	*Not detected*	*Not detected*
	NHA E6/E7 hTert	336 ± 96	353 ± 107
IL-8	T98G	1747 ± 441	2438 ± 537
	SF268	2179 ± 180	4615 ± 467
	SF295	3076 ± 479	7702 ± 481
	NHA E6/E7 hTert	4307 ± 181	2257 ± 415

**Figure 4:**
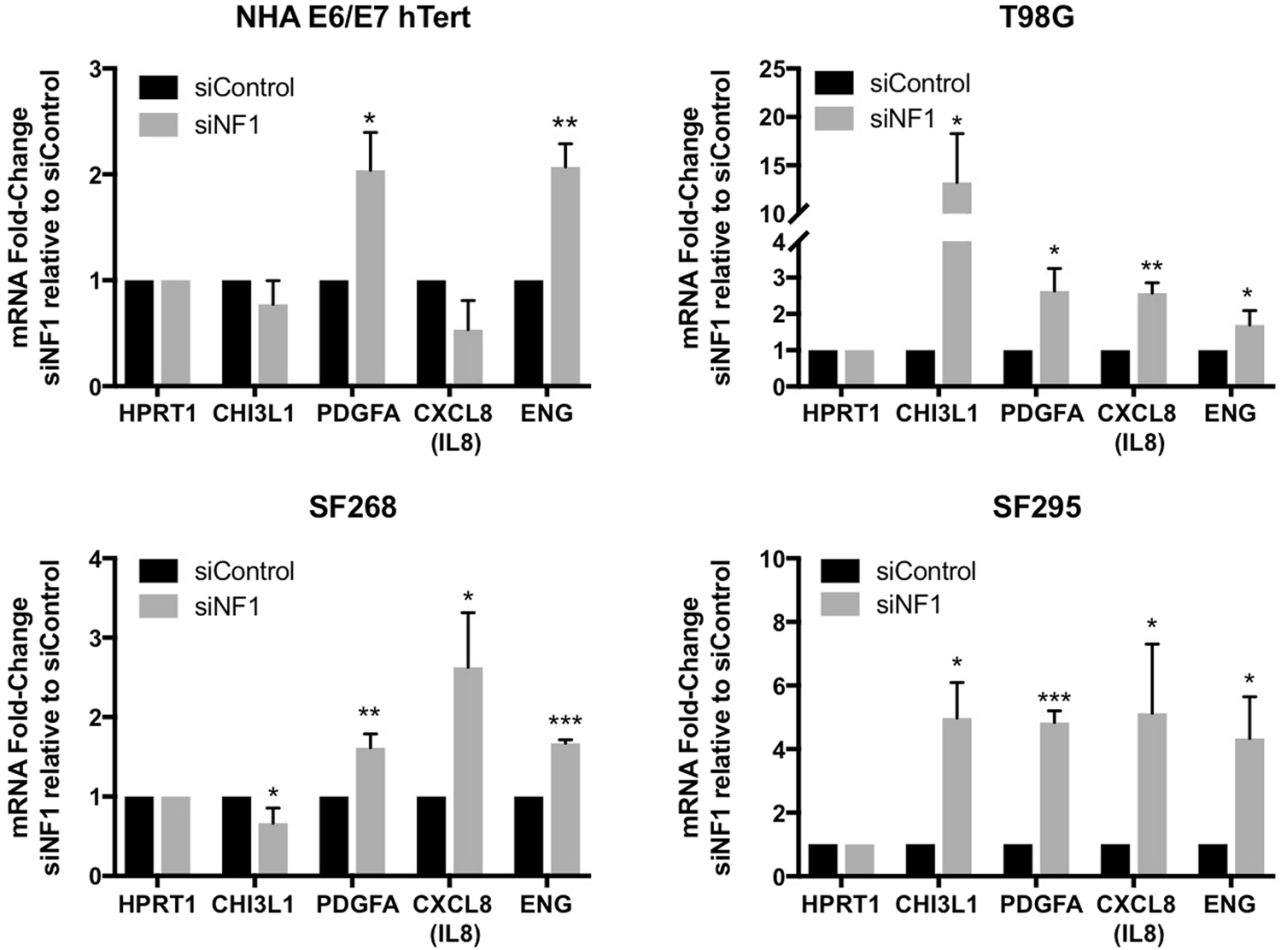
Quantitative RT-PCR for transcriptional induction or repression of the *NF1* regulated factors that were identified by cytokine array in siNF1-treated cell lines.

To determine whether there is transcriptional upregulation of the factors that we identified in the cytokine arrays and ELISA, we performed quantitative RT-PCR for *CHI3L1*, *PDGFA*, *ENG* and *CXCL8/IL8* on all four cell lines transfected with siNF1 versus control siRNA (Fig. 4). In agreement with the ELISA data where secreted cytokines/chemokines were increased, we identified transcriptional upregulation of the following genes upon *NF1* knockdown: *PDGFA* in NHA-E6/E7-hTert, T98G, and SF295 cells; *CHI3L1* in T98G and SF295 cells; *ENG* in T98G cells, and *CXCL8/IL8* in T98G, SF268, and SF295 cells. Also consistent with the reductions in secreted protein levels on ELISA, we observed a trend towards reduced *CHI3L1* and *CXCL8/IL8* in NHA-E6/E7-hTert cells, though this did not reach statistical significance. We observed transcriptional changes in some factors that were not detectable by ELSA in the same cell line, specifically induction of *ENG* in SF268 and SF295 and reduction of *CHI3L1* in SF268, which may reflect the higher sensitivity of quantitative RT-PCR. In only two instances, significant changes in the mRNA transcript levels were observed without a corresponding change in secreted protein: *PDGFA* was induced in SF268 without increased secretion, and *ENG* was induced in NHA-E6/E7-hTert without increased secretion. With these two exceptions, the qPCR data was fully concordant with the ELISA results, suggesting that neurofibromin loss could influence the glioma cell secreted protein profile through regulation of transcription. A summary of the significant changes across three testing modalities (i.e. cytokine array, ELISA, and quantitative RT-PCR) is provided as Supplemental Figure S3.

**Figure 5:**
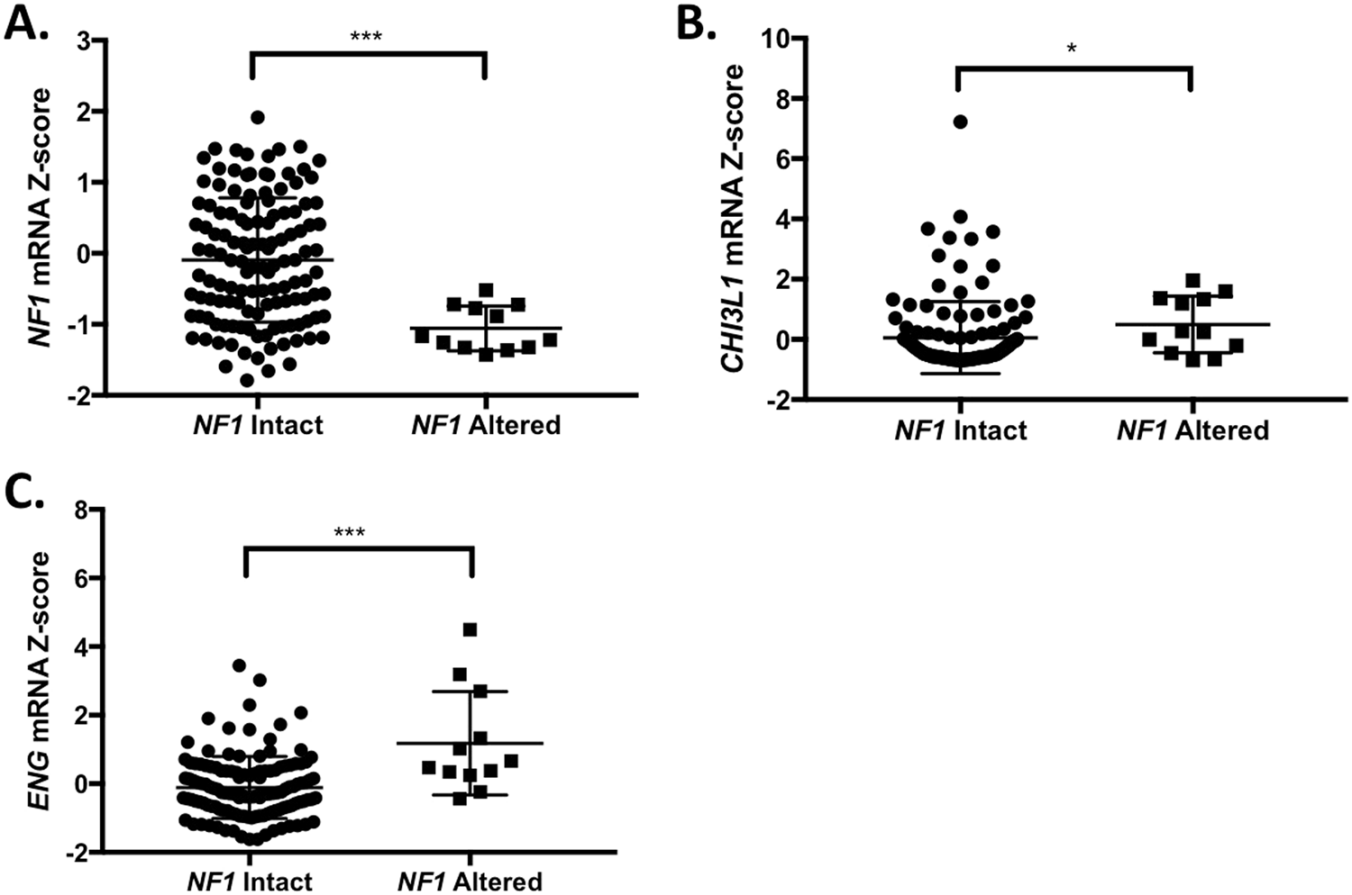
TCGA analysis of gene expression levels in *NF1* altered GBM. Z-scores of the mRNA expression values for (**A**) *NF1*, (**B**) *CHI3L1*, and (**C**) *ENG* are shown.

We turned to The Cancer Genome Atlas (TCGA) data to determine the *in vivo* relevance of our findings in human tumor samples. TCGA glioblastoma cases were classified into two categories: those with an alteration of the *NF1* gene (i.e. mutation, deletion, or mRNA downregulation, N = 12) or no evidence of a deleterious *NF1* alteration (N = 129). Cases in the *NF1*-altered group showed a significantly lower *NF1* mRNA z-score compared to the *NF1* intact group, confirming that these categories differ in their levels of *NF1* mRNA expression (Fig. 5A). The *NF1*-altered group of tumors showed significantly higher expression of *CHI3L1* and *ENG* (Fig. 5B,C), while the *PDGFA* and *CXCL8/IL8* RNA levels were not significantly altered between the two groups (Supplemental Figure S4). Taken together with the *in vitro* findings, these data raise the possibility an *in vivo* link between loss of neurofibromin and increased expression of CHI3L1 and ENG, two factors that could reflect GBM mesenchymal identity.

## Discussion

Mutations and structural alterations of the *NF1* tumor suppressor gene are common in GBM, but the significance of these genetic alterations for tumor behavior is not fully understood. *NF1* alterations are enriched in the mesenchymal gene expression subgroup of GBM, and recent studies suggest that this gene expression subclass is influenced at least partly by the tumor microenvironment^11,23^. This raises the question of how neurofibromin deficiency in neoplastic astrocytes could influence the microenvironment. To begin to understand this role for *NF1* loss in GBM, we performed an *in vitro* study of glioma cell lines and compared the results to non-transformed immortalized normal human astrocytes. These NHA-E6/E7-hTert cells have been extensively studied, grow rapidly, and are readily transfected, circumventing some of the technical limitations of non-immortalized primary human astrocytes. *NF1* knockdown under our experimental conditions resulted in increased secretion of surprisingly few cytokines and chemokines in the glioma cell lines. We found evidence for an association between *NF1* loss and transcriptional upregulation and secretion of CHI3L1 and ENG, two markers of mesenchymal lineage cell types that are expressed in a subset of GBM. We also identified transcriptional upregulation of *PDGFA* and *CXCL8/IL8* and corresponding increased secretion of PDGF-AA and IL-8, two factors that can influence tumor inflammatory cell content and vascular proliferation. Interestingly, we observed that in NHA-E6/E7-hTert cells, secreted IL-8 and CHI3L1 levels were reduced by *NF1* knockdown, and secreted ENG was unchanged, suggesting that the effects of *NF1* loss on CHI3L1, IL-8, and ENG secretion is different in transformed versus non-transformed astrocytes. NHA-E6/E7-hTert cells are, however, different from primary astrocytes, so further study is required to confirm this relationship.

Chitinase-3-like protein 1 (CHI3L1; also called YKL-40) is a secreted chitin-binding glycoprotein that lacks chitinase activity^24^. CHI3L1 is expressed in a subset of gliomas, and an *in vitro* study has implicated CHI3L1 in promoting glioma cell invasion and survival^25^. A number of immune and mesenchymal cell types express CHI3L1 during cellular differentiation or pathologic inflammatory conditions, but astrocytes have also been shown to produce CHI3L1 *in vivo* under the control of proinflammatory cytokines produced by macrophages, and in the setting of glial neoplasms^26,27^. Interestingly, CHI3L1 is an immunophenotypic marker of the mesenchymal gene expression subclass of GBM^28^. This study is the first suggestion, to our knowledge, of a direct link between *NF1* loss in glioma and production of CHI3L1 by neoplastic cells. Since CHI3L1 is implicated as a driver of some aspects of mesenchymal behavior in GBM, our study raises the possibility of a mechanistic link between *NF1* and mesenchymal phenotype, mediated in part by CHI3L1 that is produced by the neoplastic glioma cells.

Endoglin (ENG; also called CD105) is a transmembrane protein component of the transforming growth factor beta (TGF-β) receptor complex that is expressed in approximately 40% of primary glioblastoma samples, but not in secondary GBM or normal brain^29^. ENG expression is controlled, at least in part, by TGF-β signaling and contributes to mesodermal differentiation in primary GBM^30^. As with CHI3L1, the results from our study raise the possibility of a direct link between *NF1* loss and mesenchymal identity *in vitro*. Interestingly, the relationship between GBM *NF1* status and increased expression of CHI3L1 and ENG was preserved in TCGA data, suggesting that there is *in vivo* relevance for these findings. The mechanistic significance of secreted ENG in glioma is not clear. In mouse models of skin carcinogenesis, membrane-associated ENG suppresses H-Ras mediated transformation^31^. Shedding of the membrane-associated ENG is associated with increased TGF-β signaling and progression to spindle morphology in squamous cell carcinoma^32^. The increase in secreted ENG that we observed in *NF1*-deficient T98G cells might therefore reflect an increase in TGF-β signaling, a pathway that is implicated in mesenchymal transition in GBM^33^.

Interleukin-8 is expressed in GBM and mostly has pro-angiogenic effects, but is also implicated in neutrophil chemotaxis and autocrine growth-promoting effects^34^. In our study, *NF1* knockdown was associated with increased transcription and secretion of IL-8 in 2 of 3 glioma cell lines. However, we did not observe a relationship between GBM *NF1* status and *CXCL8/IL8* mRNA levels *in vivo*, as assessed by TCGA analysis. Similarly, although we identified increased secretion of PDGF-AA in two glioma cell lines as well as immortalized normal human astrocytes upon *NF1* knockdown, this relationship was not preserved in the TCGA data.

Our study has a number of limitations. First, we assessed a small number of cell lines using *in vitro* approaches, so our findings do not reflect the complexity of the tumor/microenvironment interaction that occurs *in vivo*. Second, we did not seek to define the precise mechanisms by which *NF1* deficiency leads to increased secretion of such a small number of cytokines and chemokines, beyond suggesting that part of the mechanism is by transcriptional upregulation. Further studies are required to define a signaling pathway responsible for induction of the factors that we found in this work. Third, some of the relationships identified in our study are not supported by *in vivo* data from TCGA – specifically for PDGF-AA and IL-8. Part of this limitation can be attributed to tissue culture artifacts, but it is also likely related to the small number of *NF1-*deficient GBM samples in the database. Finally, although the siRNA constructs that we used are modified for maximum specificity, rescue experiments would be required to prove the findings are due to an on-target (i.e. neurofibromin-specific) effect. However, we note that the siRNA constructs that we employed have been validated and used in previous studies, we identified commonalities across different cell lines, and we used TCGA data to support the findings^35,36^. Therefore, we consider it unlikely for our findings to be solely an off-target phenomenon.

As clinical next-generation sequencing for brain tumors becomes more common, more *NF1*-altered gliomas will be identified, and the identification of such tumors will be critical for further study. Our data suggest that *NF1* loss could directly alter the regulation and expression of factors that contribute to cellular mesenchymal identity and the tumor/microenvironment interaction.

## Electronic supplementary material

Supplementary Information
