# Supplementary material for: Neurofibromin knockdown in glioma cell lines is associated with changes in cytokine and chemokine secretion in vitro
Source: Sci Rep. 2018 Apr 11;8:5805. doi: 10.1038/s41598-018-24046-2 (PMC5895785; doi:10.1038/s41598-018-24046-2)

Supplementary Information for:

Neurofibromin knockdown in glioma cell lines is associated with changes in cytokine and chemokine secretion *in vitro*

Matthew D. Wood[1, 3], Joydeep Mukherjee[2], and Russell O. Pieper[2*]

[1]Department of Pathology, Division of Neuropathology, University of California San Francisco, San Francisco, CA

[2]Department of Neurological Surgery, University of California San Francisco, San Francisco CA

[3]Current address: Department of Pathology, Oregon Health & Science University, Portland, OR

Corresponding Author:

Dr. Russell O. Pieper
University of California San Francisco
1450 Third Street
San Francisco, CA 94158
Phone: 415-502-7132
Fax: 415-502-7126
E-mail: Russ.Pieper@UCSF.edu

Supplemental Figure S1: Expanded Western blot images for siRNA-treated cell lines used in this study. In order to enable the analysis of multiple protein from a single replicate, membranes were stained with Ponceau S and trimmed into strips. The portion from 100 kDa and higher was used for NF1 Western blotting. The portion from 25 kDa to 50 kDa was used first for P-Erk Western, then the membrane was stripped using Restore Western stripping buffer reagent (ThermoFisher) and re-probed for total ERK. Finally, the 25-50 kDa portion was stripped a second time and re-probed for GAPDH. All panels for NHA E6/E7-hTert and SF 268 came from one membrane, and all images for T98G and SF295 came from another membrane, which was treated and blotted using identical conditions. The images for all three proteins are always from a single experiment. For the P-Erk panels, * marks the band of interest; the < 37 kDa molecular weight band does not react with total ERK, and is nonspecific.

Supplemental Figure S2: Complete cytokine array images (A-C) and quantitation of cytokine array data (D-F) for NHA E6/E7 hTert, SF268, and SF295 cell lines. Array images and most of the quantitations are from 2 minute exposures.

Supplemental Figure S3: Summary of cytokine array, ELISA, and qPCR data for PDGF-AA, CHI3L1, ENG, and IL-8 across four cell lines. For ELISA and qPCR data, statistically significant increases are in green text, and statistically significant decreases are in red text. For cytokine array data, changes greater than 1.5-fold are highlighted in green (increase) or red (decrease).

Supplemental Figure S4: TCGA data for *PDGFA* and *CXCL8/IL8* mRNA expression levels in TCGA GBM samples with or without *NF1* alteration.

Supplemental Figure S1

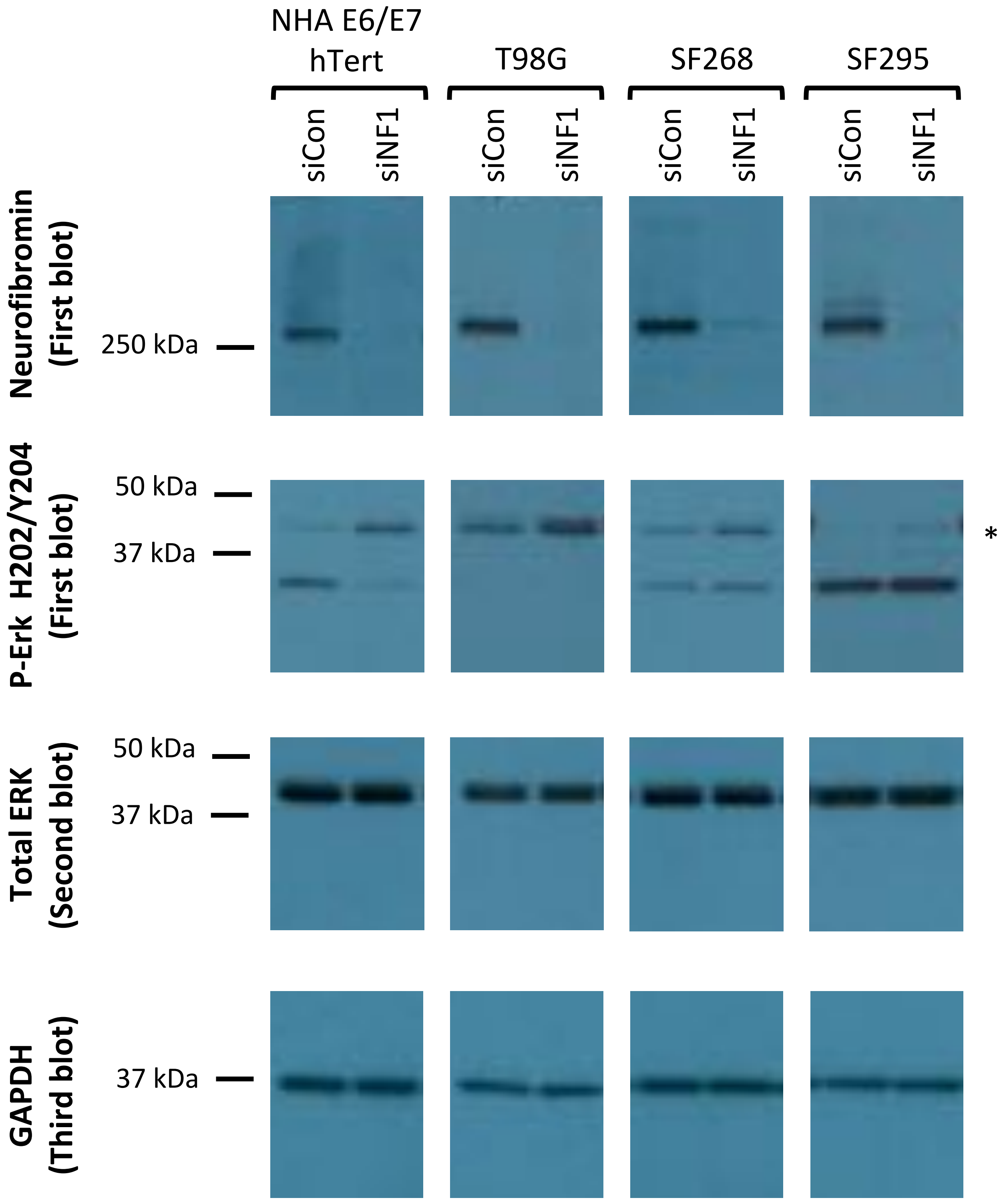

Supplemental Figure S2

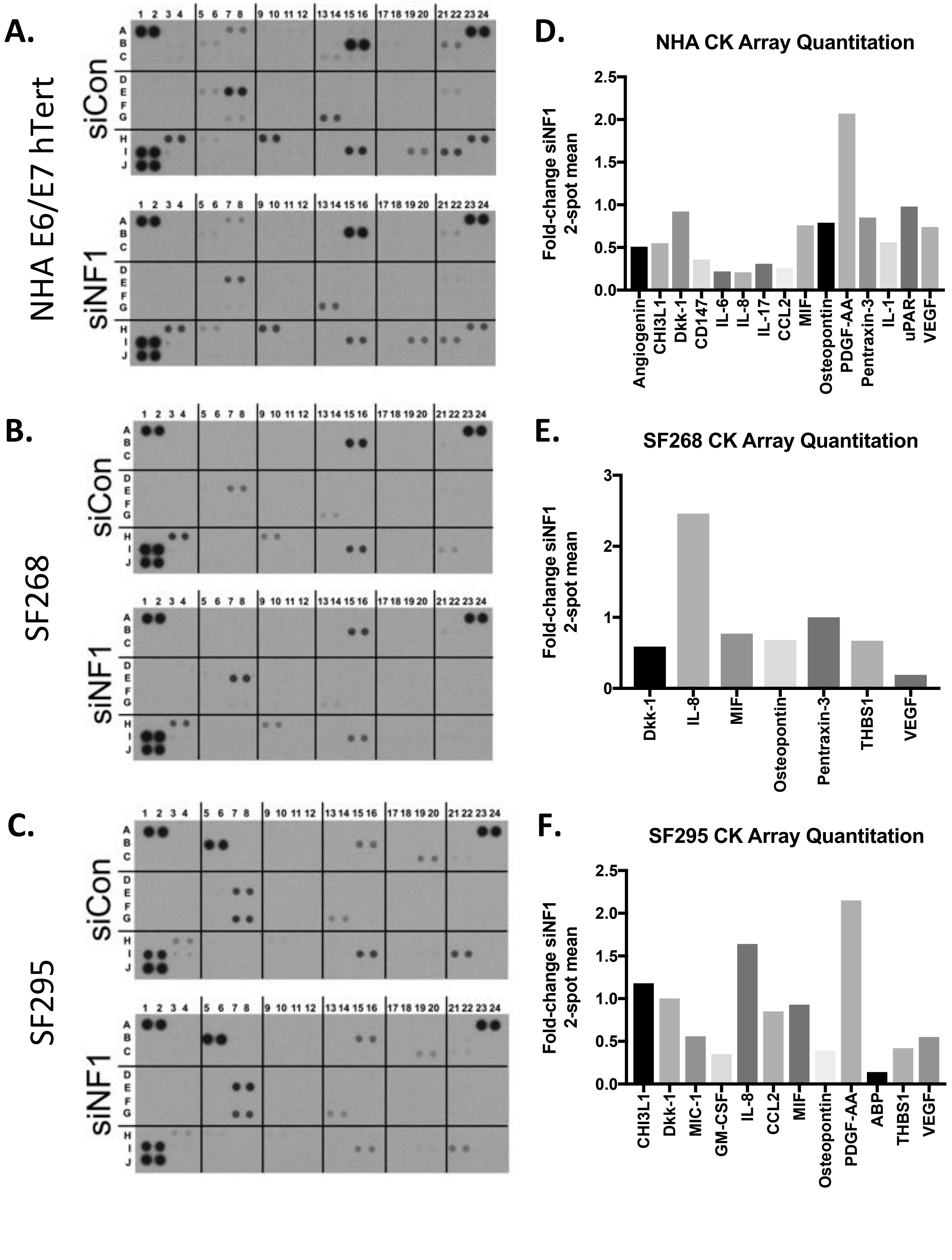

Supplemental Figure S3

		NHA E6/E7 hTert	T98G	SF268	SF295
PDGF-AA	CKA	**2.07**	**4.89**	Not detected	**2.15**
	ELISA	**2.78**	**3.03**	0.94	**3.86**
	qPCR	**2.04**	**2.63**	**1.62**	**4.83**
CHI3L1	CKA	**0.55**	**7.07**	Not detected	1.18
	ELISA	**0.36**	**4.89**	Not detected	**1.55**
	qPCR	0.77	**13.20**	**0.67**	**4.98**
IL-8	CKA	**0.21**	1.09	**2.46**	**1.64**
	ELISA	**0.54**	**1.34**	**1.87**	**2.67**
	qPCR	0.53	**2.53**	**2.63**	**5.12**
ENG	CKA	Not detected	**2.8**	Not detected	Not detected
	ELISA	1.04	**1.33**	Not detected	Not detected
	qPCR	**2.07**	**1.70**	**1.67**	**4.34**

Supplemental Figure S4

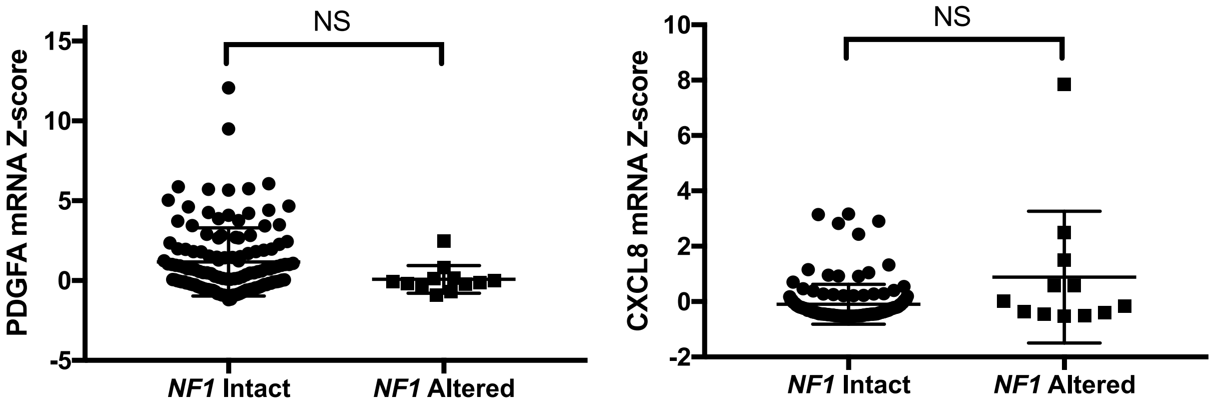

Supplement: Supplementary file 1 — Supplementary Information [file 41598_2018_24046_MOESM1_ESM.pdf]
